# Examination of an in vitro methodology to evaluate the biomechanical performance of nucleus augmentation in axial compression

**DOI:** 10.1177/0954411917752027

**Published:** 2018-01-13

**Authors:** Sebastien NF Sikora, Danielle E Miles, Sami Tarsuslugil, Marlène Mengoni, Ruth K Wilcox

**Affiliations:** 1 Institute of Medical and Biological Engineering, University of Leeds, Leeds, UK; 2 School of Chemistry, University of Leeds, Leeds, UK

**Keywords:** Spine, intervertebral disc, nucleus augmentation, mechanical testing, finite element analysis

## Abstract

Intervertebral disc degeneration is one of the leading causes of back pain, but treatment options remain limited. Recently, there have been advances in the development of biomaterials for nucleus augmentation; however, the testing of such materials preclinically has proved challenging. The aim of this study was to develop methods for fabricating and testing bone-disc-bone specimens in vitro for examining the performance of nucleus augmentation procedures. Control, nucleotomy and treated intervertebral disc specimens were fabricated and tested under static load. The nucleus was removed from nucleotomy specimens using a trans-endplate approach with a bone plug used to restore bony integrity. Specimen-specific finite element models were developed to elucidate the reasons for the variations observed between control specimens. Although the computational models predicted a statistically significant difference between the healthy and nucleotomy groups, the differences found experimentally were not significantly different. This is likely due to variations in the material properties, hydration and level of annular collapse. The deformation of the bone was also found to be non-negligible. The study provides a framework for the development of testing protocols for nucleus augmentation materials and highlights the need to control disc hydration and the length of bone retained to reduce inter-specimen variability.

## Introduction

Four out of five adults will suffer low back pain during their lifetime, with many going on to suffer further acute episodes, imposing a high economic and social burden on society.^1–3^ Low back pain is strongly associated with degeneration of the intervertebral discs, which causes a loss of hydration of the tissue and reduces its swelling pressure. Current surgical treatments are highly invasive and have low long-term success rates. Alternative solutions include nucleus augmentation; however, as yet there has not been widespread clinical uptake of this technology, partly due to reported complications such as implant extrusion.^4^

An increasing number of biomaterials are being investigated or specifically developed for nucleus augmentation applications,^5–7^ but the methods available to preclinically test or compare these materials in a realistic in vitro environment have been limited. One challenge is to represent the biomechanical behaviour of the degenerated disc in vitro without compromising the integrity of the annulus fibrosis, so that the only damage to the annulus is caused by the simulated nucleus augmentation procedure itself. Previous studies have simulated degeneration by enzymatic digestion^7–9^ or by physical removal of the nucleus tissue using a trans-endplate nucleotomy.^5,10–13^ The former requires a lengthy incubation period and is difficult to standardise, while the latter requires drilling through the adjacent vertebral bone and endplate, inevitably compromising the nucleus boundary and providing another route of escape for any injected biomaterial.

A number of groups have developed increasingly sophisticated cyclic loading methodologies for the evaluation of both the natural disc and the tissue following treatment.^5,7,11–17^ These approaches better mimic the in vitro situation by testing the disc within a fluid and enabling flow through the endplates and/or annulus. However, the lengthy testing protocols and relatively small differences in stiffness observed even between positive and negative control groups (e.g. native tissue vs a nucleotomy)^10,11^ mean that the use of such testing to compare between candidate nucleus augmentation products is likely to be limited. One of the major issues is that there tend to be large variances in the measured outputs, which potentially mask any effects of treatment.^10,13^ Therefore, there remains a need to develop in vitro testing methods that can be used to examine and compare the performance of nucleus augmentation methods in a realistic environment.

The aim of this study was to develop methods for fabricating standardised control, nucleotomy and treated intervertebral disc specimens and examine the reasons for the variations that occur between them, with the ultimate aim of developing methods for examining the biomechanical performance of nucleus augmentation procedures. In this study, a simple static loading approach was considered as a potential method for screening materials before lengthier cyclic testing. The method was applied to examine the performance of a recently developed self-assembling peptide:glycosaminoglycan hybrid hydrogel (peptide:GAG hydrogel).^6^ Finite element (FE) models of the specimens were used to examine the reasons for the variations observed between specimens and propose further improvements to the approach.

## Methods

### Experimental methods

#### Specimen preparation

Bovine tails harvested from cows under the age of 30 months were obtained from a local abattoir on the day of kill. Bovine tails were selected due to the ease with which animals from within a narrow age range were available and due to the absence of facet joints meaning all load transfer occurs only through the intervertebral disc. The sections of each tail comprising the four most anterior discs (C1–C5) were isolated and soft tissues were carefully removed, avoiding damage to the fat-capsule surrounding each disc. The C1–C5 tail sections were sealed into plastic bags before being frozen and stored at −80 °C.

**Figure 1.**
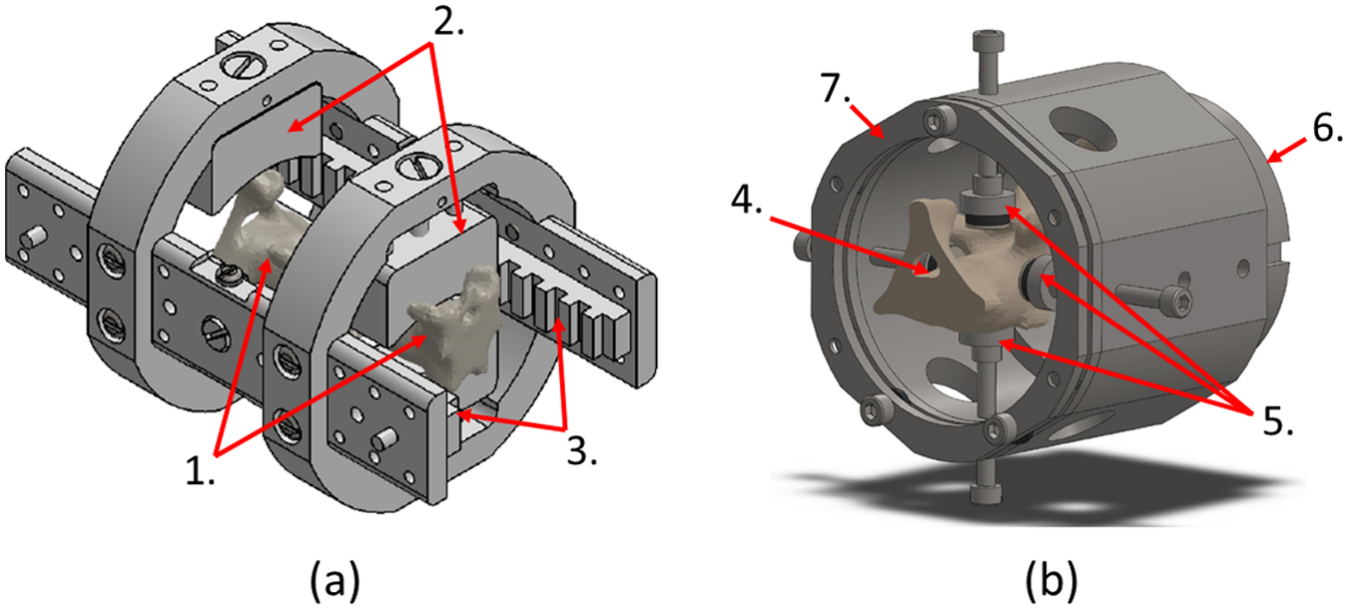
Bespoke measuring (a) and cutting (b) jigs used in specimen preparation. Measuring jig axially clamps specimen (1) between locating jaws (2). Following µCT imaging, indications of cutting-plane identified on µCT images transferred to specimen via reference to radio-opaque marker strips (3). Cutting jig axially clamps specimen (4) via adjustable supports (5) and base clamp (6). A hacksaw blade is then introduced between the jig body and removable guide plate (7), which ensures cuts are perpendicular to the longitudinal tail axis.

On the day of specimen preparation, the tail sections were segmented into individual functional spinal units (FSU). First, each tail section was mounted on a custom measuring jig (Figure 1(a)) and imaged using micro-computed tomography (µCT) at a voxel size of 73.4 μm (microCT100, Scanco Medical AG, Switzerland). The µCT images were used to identify parallel planes through adjacent vertebrae perpendicular to the craniocaudal axis, 15 mm from the adjacent vertebral endplates, which were then marked onto the tail sections using the measuring jig. The tail sections were then transferred to a custom cutting jig (Figure 1(b)) designed to facilitate plano-parallel cuts through the tail section perpendicular to the craniocaudal axis. From each C1–C5 tail section, only the first three levels (C1–C4) were selected to reduce variability due to spinal level. The inferior bone surface of each FSU was then potted into poly(methyl methacrylate) (PMMA) end-caps sized to fit custom compression test fixtures.

**Figure 2.**
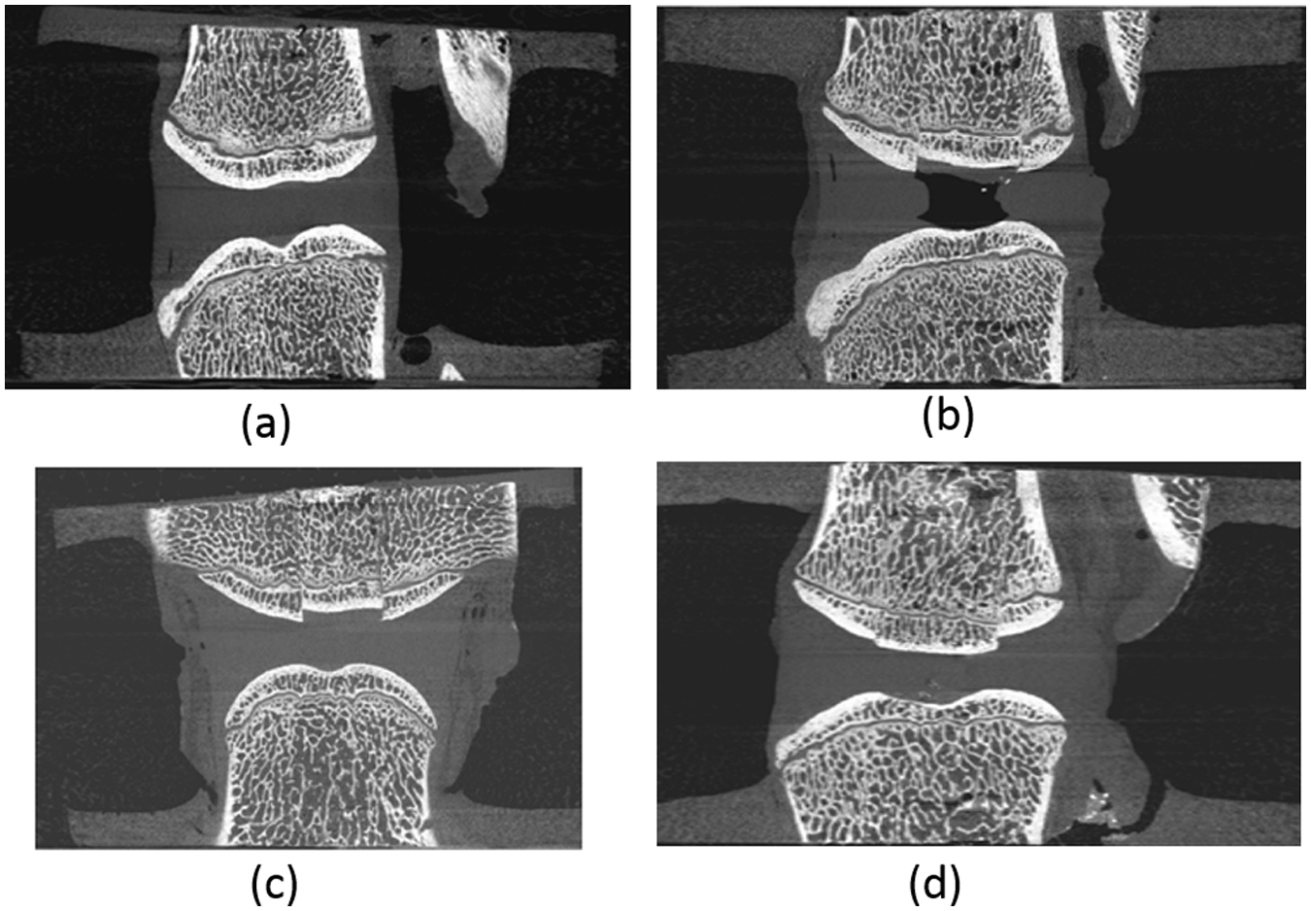
µCT transverse view of the four tested groups: (a) CON, (b) NR, (c) BP and (d) PEP.

**Figure 3.**
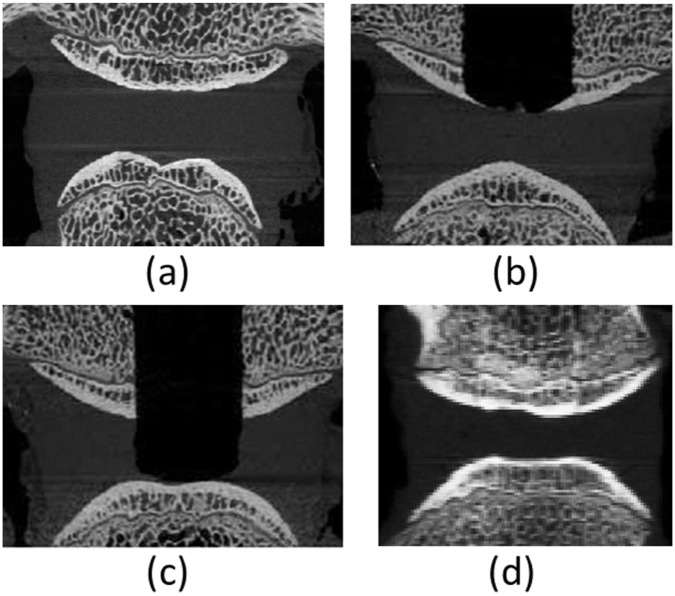
Nucleotomy process: (a) CON specimen, (b) BP specimen before insertion of bone allograft into the superior vertebra, (c) NR specimen before insertion of bone allograft and (d) PEP specimen ready for testing, that is, following insertion of bone allograft and injection of hydrogel.

The 24 individual FSUs harvested from eight tails and prepared in this manner were then divided between four test groups (Figure 2) – control (CON), bone plug (BP), nucleus removed (NR) and peptide:GAG hydrogel augmented (PEP) – and distributed such that there was no preponderance of any particular level or tail in any one group:

1. The control (CON) group (Figure 2(a)) served as an unmodified control representing the behaviour of a healthy non-degenerated disc.
2. The NR group (Figure 2(b)) served as nucleotomy control. The nucleotomy procedure was intended to allow peptide:GAG hydrogel augmentation of the treated specimens without over-pressurisation of the disc and possible damage. A trans-endplate nucleotomy was undertaken (Figure 3(a)–(c)) where a 10-mm-diameter central region of the nucleus, as identified on µCT imaging, was removed by drilling through the superior vertebra and endplate, avoiding damage to the inferior endplate. To restore the nucleus boundary following nucleotomy, an oversized bone allograft produced by excising the central superior vertebral bone from an additional FSU of similar level was inserted into the hole in the superior vertebral bone to create a push-fit graft (Figure 3(d)).
3. In a complementary manner, the BP group (Figure 2(c)) served as a further control group to allow isolation of any effects resulting from the modification of the superior vertebral bone in groups that included nucleotomy. These were fabricated in the same manner as those specimens in the NR group, with the exception that the bone drill was stopped short of the nucleus following trans-endplate approach, and the nucleus was not excised (Figure 3(a) and (b)).
4. The peptide-augmented (PEP) group (Figure 2(d)) served to represent a disc repaired via peptide:GAG hydrogel augmentation (see next section), prepared in the same manner as the NR group.

Following group-specific preparation, the superior bone surface of each FSU was potted into PMMA end-caps, keeping each FSU in their natural position.

To assist in maintaining the natural hydration state of the tissue as far as possible, in between operations the specimens were wrapped in tissue paper moistened with physiological buffered saline (PBS), sealed into plastic bags and returned to storage at −80 °C. Before mechanical test or hydrogel augmentation, the bagged specimens were stored at 4 °C until completely defrosted, with the PBS-soaked tissue removed only immediately prior to the test. Due to the short duration of the mechanical test protocol, specimens were not actively hydrated while under test.

#### Peptide:GAG hydrogel augmentation

An injectable peptide:GAG hydrogel with rapid self-assembly was used in this study.^6^ It was developed to form a gel in situ with mechanical properties comparable to the native tissue. This specifically designed hybrid hydrogel can be injected through a narrow gauge needle and the presence of GAGs mimics the ability of the native tissue to draw in water.

Prior to mechanical testing, specimens in the PEP group underwent augmentation with the peptide:GAG hydrogel. Specimens were imaged using µCT while frozen to determine the volume of the void within each de-nucleated disc and to identify appropriate needle placement location and angle. Determination of the void volume provided a target volume for injection into each disc and enabled calculation of the quantities of peptide and GAG that would result in the correct ratio. Two 29G needles were inserted through the annulus into the post-nucleotomy void. Calculated quantities of GAG solution and then peptide solution were injected in turn via one needle while the second acted as a vent to reduce injection back-pressure. Following augmentation, both needles were removed.

#### Mechanical testing

Immediately prior to testing, the specimens were imaged using µCT. These images were used both to examine the results of the peptide:GAG hydrogel augmentation of the specimens in the PEP group and to enable the generation of specimen-specific FE models of each specimen in its pre-loaded state.

Quasi-static axial compression of all specimens was conducted using a materials testing machine (ElectroPuls E10000, Instron, USA) with specimens in their relaxed position following thawing. To ensure zero transversal pre-load, the specimens were mounted to polyoxymethylene fixtures fixed to the machine cross-head via two stacked linear bearings that allowed free translations perpendicular to the direction of the applied displacement. The specimens were tested up to a maximum load of 2.1 kN (chosen according to the upper bound given by the ISO standard for wear testing of lumbar total disc replacement devices)^18^ at a rate of 1 mm/min following a pre-load of 10 N. Natural tissues often exhibit mechanical behaviour sensitive to the immediate load history, and for this reason, an initial mechanical preconditioning protocol is often applied prior to testing. However, due to the requirement to maintain specimen hydration state and avoid the displacement of fluid from the disc structure, no initial preconditioning was conducted in this case.

### Computational methods

**Figure 4.**
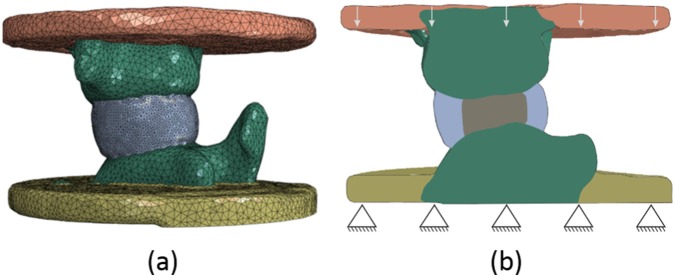
Specimen-specific finite element model of one of the CON specimens showing (a) the geometry and mesh, and (b) the boundary conditions.

Image-based computational models of the CON, BP and NR groups were produced to examine the underlying variability of the specimens across the control groups. The µCT images were scaled to 8-bit greyscale and specimen-specific geometries were derived using image processing tools in Simpleware ScanIP (Synopsys, Mountain View, USA) with images down-sampled to an isotropic 0.5 mm resolution. As soft tissues cannot be differentiated in the µCT images, a consistent protocol was developed to create the annulus and nucleus regions of the disc based on an annulus:nucleus diameter ratio of 2:1 as measured in a parallel study (Figure 4). FE models were produced using the segmented geometry and underlying greyscale, using a homogeneous linear tetrahedral mesh with a size based on previous studies.^19,20^ Given models were built from µCT images of FSU in their PMMA end-caps, relative position of both vertebrae in each FSU was modelled as positioned prior to mechanical testing.

Material properties were assigned as shown in Table 1, with a varying bone elastic modulus linearly dependent on the image greyscale, anisotropic hyperelastic annulus assuming two oblique/counter-oblique fibre orientations at 20° to the sagittal plane,^21^ and isotropic hyperelastic incompressible nucleus for the specimens without nucleotomy. Boundary conditions replicating the experimental tests were applied. The lower surface of the distal end-cap was clamped, and an axial translation, of the same magnitude as the corresponding displacement reached in the in vitro model, was applied to the upper surface of the cranial end-cap while all other translations were kept free and all rotations were restricted. All FE analyses were non-linear quasi-static and run in parallel with Abaqus 6.14 (Simulia, Dassault Système, USA).

**Table 1.** Material models and parameters used in the finite element models.

Tissue type	Material model	Material parametervalues	Reference for materialparameters
Annulus fibrosus	Anisotropic non-linearelasticity (Holzapfel model)	C_10_ = 0.32 MPa K = 2200 MPa k_1_ = 2.45 MPa k_2_ = 2.11	Monaco et al.^22^ (C_10_), compressibility of water (K), Cortes and Elliott^23^ (k_1_ and k_2_)
Nucleus pulposus	Non-linear incompressibleelasticity (Mooney–Rivlin model)	C_10_ = 0.07 MPa C_01_ = 0.02 MPa	Adam et al.^24^
Bone	Greyscale (GS_ele_) basedlinear elasticity	E_ele_ = linearly varying with GS_ele_ *ν* = 0.3	Wijayathunga et al.^25^
PMMA cement	Linear elasticity	E = 1.5 GPa *ν* = 0.3	Tarsuslugil et al.^26^

PMMA: poly(methyl methacrylate).

A sensitivity study on the bone rigidity was performed by examining two additional models where the elastic modulus of the bone was altered to be homogeneous across the whole vertebral region. In one, the modulus was based on the greyscale average of all vertebrae, in the other, the bone was considered as being much more rigid than the intervertebral disc, using a homogeneous Young’s modulus similar to that used for the PMMA cement.

### Data post processing

Force/displacement data were extracted for each specimen directly from the material testing machine outputs for the experimental tests and using the postPro4Abq toolbox for the FE models.^27^ All experimental and computational specimens showed a force/displacement behaviour with an initial toe region and a final linear zone. To normalise the data, the force/displacement data were transformed into effective stress/engineering strain using specimen-specific approximate cross-sectional area and disc height from the µCT 3D reconstructions. A custom-written algorithm was used to determine the gradients of a continuous tri-linear fit, using a non-linear least-square method in MATLAB (R2014b, The Mathworks Inc., Natick, MA, USA), defining three effective Young’s moduli (EYM) values (initial EYM, transition EYM and linear EYM), and the two transition strains between the three zones. Pearson’s coefficients and root mean square errors were used to assess the goodness of fit for the experimental data. For the CON group, the contribution of the different components of the FSU (vertebrae and intervertebral disc) to the overall apparent strains were also determined from the FE models.

Values from the tri-linear stress/strain fit were compared between groups with either analysis of variance (ANOVA) tests and post hoc pairwise t-tests or Kruskal–Wallis tests (no post hoc analysis was necessary) after the normal nature of the data was assessed with a Shapiro test. The same values were compared between experimental and computational models or between the sensitivity study results with a least-squares linear regression and concordance correlation coefficients. Finally, the variance of the paired computational and experimental values were compared with an F-test within each group.

All statistical analyses were performed with R.3.1.2 (R foundation for statistical computing) and statistical significance was set at p < 0.05.

## Results

The data associated with this article (mechanical testing, µCT images, Abaqus input files and post-processed data) are openly available from the University of Leeds Data Repository.^28^

### Mechanical testing

**Figure 5.**
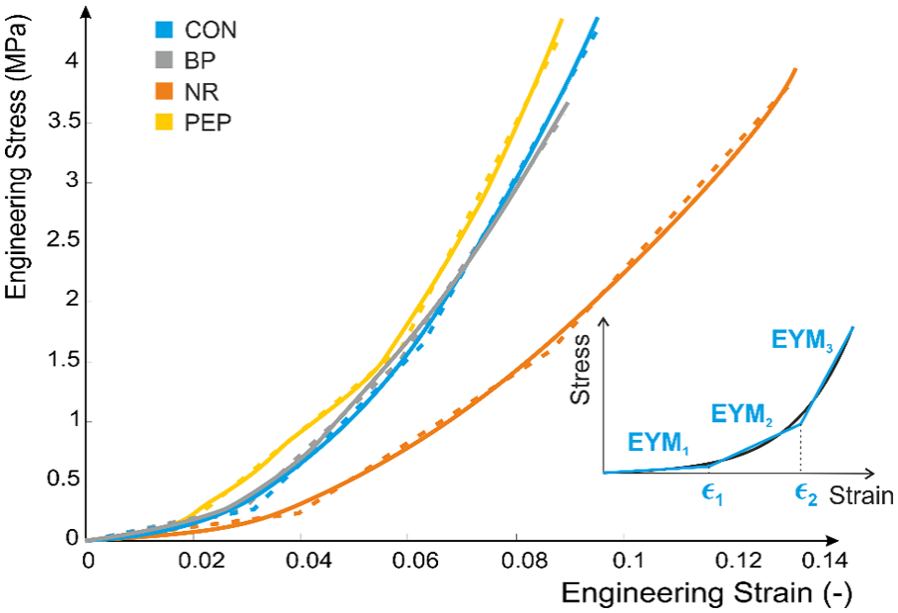
Tri-linear approximation of the experimental stress/strain behaviour, 1 specimen per group (plain line experimental data, dashed line tri-linear fit).

Stress–strain plots for all specimens in all four groups displayed the same characteristic shape, demonstrating no evidence of mechanical failure in any case. This agrees with the lack of any observed evidence of failure in either the intervertebral disc or adjacent bone. The tri-linear fit was a very good approximation of the stress/strain data before failure (Figure 5), with a root mean square error between 2.6% and 5.5% for all 24 samples and Pearson’s coefficients between 0.9987 and 0.9996.

**Figure 6.**
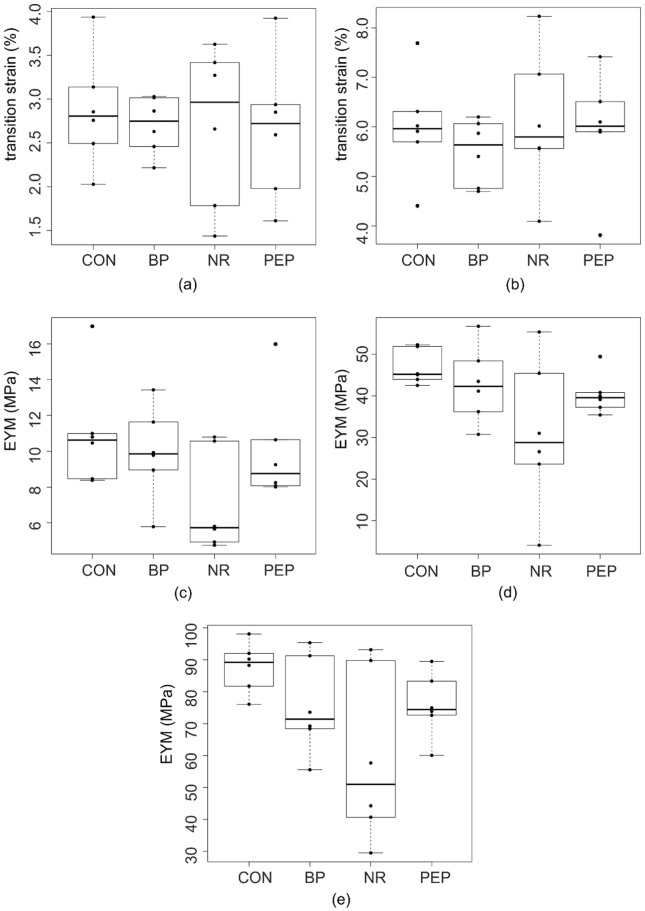
Comparison of (a) first transition strains and (b) second transition strains and three EYM values (c) initial, (d) transition and (e) linear between the four experimental groups.

No significant differences were observed in any of the EYM values or transition strains between the four groups, with large variances seen particularly in the NR group (Figure 6).

### Computational results

The range of bone elastic modulus values obtained from the element-by-element greyscale-based model was 10–910 MPa, with a volume average of 409 MPa.

No significant differences were found between both control groups (CON, BP) for any of the values of interest. The NR group exhibited transition and linear EYM values significantly lower than those of both control groups (post hoc pairwise t-test p-values < 0.05).

**Figure 7.**
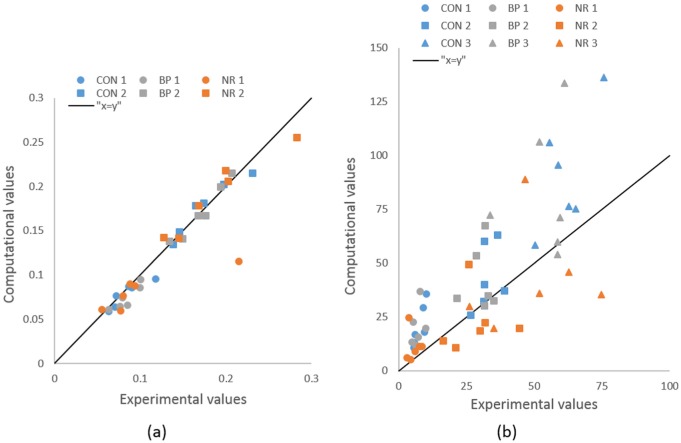
Concordance between experimental and computational mechanical values of interest: (a) transition strains and (b) effective Young’s moduli (MPa).

All computational values of interest, except transition and linear EYM in NR group, showed the same or higher variability as their experimental equivalent(p < 0.03), without significant difference in their mean values within each group (Figure 7). The computational EYM values presented good correlation with their experimental equivalent in the CON group (coefficients of determination above 0.6) but poor correlation (coefficients of determination lower than 0.2) in the other two groups. All three EYM values in three groups presented very poor concordance with their experimental equivalent (concordance correlation coefficients below 0.2). The transition strains showed high correlation (coefficients of determination above 0.7) and good concordance (concordance correlation coefficients above 0.6) between computational and experimental data for both values in all groups.

**Figure 8.**
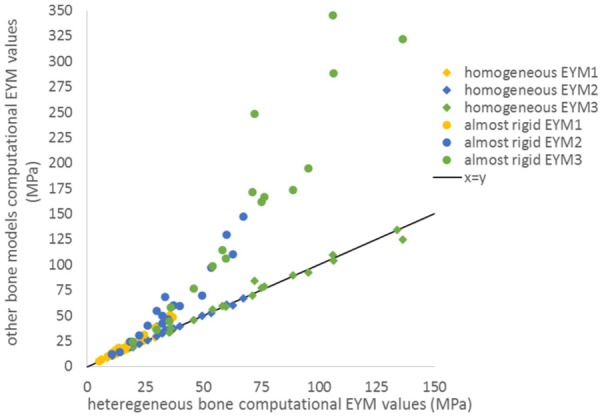
Concordance between heterogeneous bone and homogeneous or almost rigid bone for the effective Young’s moduli (all groups).

The bone sensitivity study showed that the three EYM values across the three groups were in very good concordance (CCC > 0.989) between the baseline heterogeneous model and the homogeneous bone model based on the average greyscale of the images across the 36 vertebrae (Figure 8). The transition strain values showed a good concordance (CCC = 0.840 and 0.953 for transition strain 1 and 2, respectively) as well. The concordance between the baseline model and the near-rigid homogeneous bone model was poor for all EYM values (CCC = 0.868, 0.525 and 0.295 for initial, transition and linear EYM values, respectively). The transition strains showed good concordance (CCC = 0.544 and 0.890 for transition strain 1 and 2, respectively).

In the six specimens of the CON group, the contribution of bone to the applied apparent strain varied from 1.9% to 13.2%, with an average contribution of 5.7%.

## Discussion

### Introduction

This work proposed a methodology to fabricate spinal FSU specimens to test intervertebral disc interventions in a controlled environment. The premise was that the intact (CON and BP) and nucleotomy (NR) groups would effectively provide negative and positive control groups to compare the augmented (PEP) specimens against.

The static compression testing protocol was developed to standardise the procedure as much as possible and minimise experimental variance. For example, no transversal loading was applied to the specimens so that the test was purely axial even if the specimens were not completely centred within their PMMA end-caps due to the specimen geometry or the relaxed position following thawing. In addition, the length of bone retained at each side of the disc was kept constant and the mechanical values of interest were normalised to take account of variability in disc size.

However, despite these steps, the differences between the intact and nucleotomy groups were found not to be statistically significant, and relatively large variances were observed within each group. A computational approach was therefore employed to examine the reasons for these variances and enable recommendations to be made for further improvements to the testing protocol.

The results obtained from the specimen-specific FE models, which captured only the differences in geometry and bone properties between specimens, did show a statistically significant difference in mechanical behaviour between the intact and nucleotomy groups. Therefore, the original premise appeared to be sound, but additional experimental variations occurred which were not taken into account in the computational models. The different sources of variance are discussed in more detail below.

### The effect of variance

#### Variances due to bone

The fabrication method developed in this study was designed to retain a consistent amount of bone on either side of the intervertebral disc with planar surfaces at the proximal and distal ends. From the image data, this aim was achieved to an acceptable tolerance with the depth of adjacent vertebral bone being 15.72 (1.49) mm and 15.44 (2.27) mm at the proximal and distal ends, respectively.

The bovine tail vertebrae are composed of bone of relatively low quality, with an average elastic modulus, based on the greyscale average of all vertebrae, of approximately 400 MPa. The results of the FE studies showed that some specimens exhibited non-negligible bone deformation, contributing to over 10% of the apparent strain. The results of the sensitivity tests also indicated that, although the assumption of homogeneity of the bone made little difference if average properties were used, when the bone was considered as near rigid, there was a deviation in results particularly at higher strains. This again highlights that assuming bone rigidity is far from the actual behaviour. These results confirm the necessity for the fabrication method to be precise for the bone as well as the disc as the bone cannot be considered as behaving like a rigid material with respect to the tissue of interest.

#### Variances caused by the BP

Although not clinically relevant, a trans-endplate approach was selected to remove nucleus material to avoid both the annular damage involved in a trans-annular approach and the risk of possible unwanted interactions between the peptide:GAG hydrogel and enzymatic agents typically used in chemical degradation approaches. Additionally, selection of a surgical approach was considered to give greater control over the volume of removed nucleus material.

To reduce the variance induced by the nucleotomy procedure, and to maintain more physiological boundary conditions around the nucleus, a BP was used to replace the bone removed during nucleotomy. However, it was recognised that this procedure may cause some mechanical disruption, so the BP group was introduced to assess its effect. The experimental variability of all mechanical values of interest were reproduced by the computational models for the CON and BP groups. This would suggest that for those two groups the variation between specimens is mainly due to variation in geometry and quality of the bone. Given the assumptions made about the material model parameters for the intervertebral disc tissues, good concordances between computational and equivalent experimental values were not expected. The good correlations exhibited by the CON group were, however, lost for the BP group. Given the bone contribution to the deformation can be non-negligible, this suggests that the method used to push-fit the allograft may have induced changes in the bone behaviour that were not picked by the changes of greyscale values in the down-sampled µCT images. Additionally, the disruption to the endplate may have reduced the resistance to fluid flow from the disc during loading, which again would not have been captured in the computational models.

#### Variances caused by the disc

The specimens were manipulated constantly under humid conditions to reduce dehydration during fabrication. They were kept frozen throughout the process, minimising the total number of freeze-thaw cycles. Despite these steps, practical considerations in the laboratory meant that some specimens spent somewhat longer out of the freezer than others, likely impacting on the level of hydration of the tissue tested. The FE models were built using elastic model parameters from the literature for the intervertebral disc and did not account for any fluid content or non-elastic effects likely to be present in the tissue. While this produced apparent stress/strain curves whose shape matched the experimental ones, as demonstrated by the excellent correlations and concordance in the transition strain values, the models did not take account of any effects linked to a change in hydration while testing. Some of the variation seen experimentally but not computationally is therefore likely to be due to differences in the disc tissue properties from specimen to specimen, which will be altered by their hydration level at the time of testing.

In order to focus on the standardisation of the fabrication methods, this study evaluated biomechanical performance on simple static loading rather than often used cyclic protocols.^5,7,11–15,17^ This kept the testing parameters to a minimum, avoiding variability due to the testing protocol in itself. However, it is possible that equilibrating or testing the specimens in a fluid bath or cyclically loading to bring to an equilibrium state would reduce the variability due to tissue hydration.

#### Variances in the NR group

A model of the intervertebral disc with advanced degeneration was developed with a trans-endplate nucleotomy. This process increased the variability in the biomechanical outcomes measured in this study compared to the other three tested groups. This is most likely due to the differing deformation of the annulus fibrosus into the void created in the middle of the disc. In this group, the experimental values of the EYM were not larger than those of the computational models, while the opposite was true for the other two groups. The major effect not accounted for in the computational models was the fluid content of the tissues. In the experimental model, it is likely that much of the fluid content of the intervertebral disc leaks into the void created by the nucleotomy instead of only towards the external surface of the disc, increasing the total amount of fluid loss by the disc structural components. This fluid may fill up the cavity as it gets smaller due to compression. It is therefore possible that the fluid loss into the cavity has a double effect, first modifying the annulus behaviour with respect to the apparent behaviour of a healthy disc under static compression and second conferring some incompressibility to the fluid-filled void. Neither of these effects were present in the computational model and both may therefore explain the large differences in effective moduli between computational and experimental setups, as well as the larger variability in the NR group compared to the other experimental groups.

### The nucleus-augmented group

The peptide:GAG hydrogel was designed so that gelation occurs in situ post injection through a 29G needle in order to minimise damage to the surrounding tissues, to have a similar gel stiffness to that of the natural nucleus pulposus with a short gelation time to minimise the time of the procedure and a high GAG content to mimic the healthy osmotic pressure of the nucleus.

Although previous studies have reported that needle puncture damage to the intervertebral disc can result in both instantaneous and progressive mechanical consequences, the occurrence of needle-related changes in instantaneous mechanical properties appears limited to needle diameter to disc height ratios above 0.25:1.^29,30^ Given that the 29G needle used to perform the augmentation in this case results in a needle diameter to disc height ratio of 0.074 (SD 0.012):1 for these specimens, no needle-only control group was considered necessary.

The mean apparent elastic moduli of the peptide:GAG hydrogel-augmented specimens was very close to the BP group, although the variations were smaller. This suggests the peptide perhaps fills any endplate disruption under the BP and helps restore hydration, reducing the variability seen in the BP group. The testing protocol used here demonstrates that the peptide:GAG hydrogel has the potential to restore the biomechanics of a denucleated disc towards that of a disc with natural nucleus. The next step will be to transfer the methodology developed in this study to examine the peptide:GAG hydrogel in preclinical simulations that more closely mimic the behaviour in vivo, for example, in a more physiological environment under representative cyclic loading.

## Conclusion

In conclusion, the methods presented in this study provide a framework of assessment for the development of testing protocols for nucleus augmentation materials. The combined experimental and computational results indicate that a number of steps should be taken to reduce specimen-to-specimen variation: (1) there is a need to control the length of bone retained at either side of the disc since its contribution to the strain is not negligible; (2) the removal of the BP does cause some mechanical changes to the specimen and the control should therefore be specimens where the plug has been removed and replaced, rather than intact specimens; and (3) the removal of nucleus causes an increase in the variation from specimen to specimen likely due to how much annulus collapses and probable differences in hydration of remaining tissue. Therefore, the hydration level of the specimens requires standardisation in future protocols, either through testing in a fluid bath and/or under cyclic loading. Alternatively, longitudinal tests on the same specimen could be used to compare before and after nucleotomy and intervention; however, equalisation of the hydration levels would still be required prior to each test.
